# Supplementary material for: Solutions of a three-dimensional multi-term fractional anomalous solute transport model for contamination in groundwater
Source: PLoS One. 2023 Dec 8;18(12):e0294348. doi: 10.1371/journal.pone.0294348 (PMC10707926; doi:10.1371/journal.pone.0294348)
Supplement: Attachment — Submitted filename: Review Report for PONE-D-23-26366.docx [file pone.0294348.s001.docx]

**Title: Solutions of a three-dimensional multi-term fractional anomalous solute transport model for contamination in groundwater**

In this article the authors present a meshless computational approach for simulating the three-dimensional multi-term time-fractional mobile-immobile diffusion equation in the Caputo sense. The methodology combines a stable Crank-Nicolson time-integration scheme with the definition of the Caputo derivative to discretize the problem in the temporal direction. The spatial function derivative is approximated using the inverse multiquadric radial basis function. The solution is approximated on a set of scattered or uniform nodes, resulting in a sparse and well-conditioned coefficient matrix. The study highlights the advantages of meshless method, particularly their simplicity of implementation in higher dimensions. To validate the accuracy and efficacy of the proposed method, we performed numerical simulations and compared them with analytical solutions for various test problems. These simulations were carried out on computational domains of both rectangular and non-rectangular shapes. The research highlights the potential of meshless techniques in solving complex diffusion problems and its successful applications in groundwater contamination and other relevant fields.

The paper considers an interesting problem and its solution is presented adequately. The results presented are new and appealing. The paper can be accepted for publication provided the following comments:

Comment 1:  Even though the topic is really interesting and the paper have the potential to be a nice add to the literature, I found the manuscript really difficult to read. Basically, the paper needs text editing and a re-organization of the data presented.

Comment 2:  What is the shape parameter “c"?

What are the effects of this free parameter on the solution accuracy?

Comment 3:  List some properties of the Meshless method.

Comment 4:  Describe the following in details: Radial basis function, Mobile-immobile, and solute transport model.

Comment 5:  Why the presented the technique approximate the solution on set of uniform and scattered nodes and it leads to a sparse and well-conditioned coefficient matrix.

Comment 6:  Abbreviations should be defined when use at first time in the paper.

Comment 7:  The graphical visualization need to be express in clearer way. I am trying to understand the behavior of the graphs but the given information according to my point of view is not sufficient share the source how we visualize the graphs and check their accuracy. So, paste all computational work here.

Comment 8:  The punctuations at the end of the equations are missing.
