# Supplementary material for: Solutions of a three-dimensional multi-term fractional anomalous solute transport model for contamination in groundwater
Source: PLoS One. 2023 Dec 8;18(12):e0294348. doi: 10.1371/journal.pone.0294348 (PMC10707926; doi:10.1371/journal.pone.0294348)
Supplement: Attachment — Submitted filename: PONE-D-23-26366comments.docx [file pone.0294348.s002.docx]

**Exploring Solutions for a Three-Dimensional Multi-Term Fractional Anomalous Solute Transport Model in Groundwater Contamination**

In this study, the authors introduce a meshless computational technique aimed at stimulating the three-dimensional multi-term time-fractional mobile-immobile diffusion equation in the Caputo sense. The approach involves combining a stable Crank-Nicolson time-integration scheme with the definition of the Caputo derivative to discretize the temporal aspect of the problem. The spatial derivative of the function is approximated using the inverse multiquadric RBF. This integration produces a sparse linear system of equations, leading to notable reductions in computational expenses and execution time. To gauge the accuracy of their approach, the authors compute three distinct error norms. Additionally, the method's effectiveness is showcased through its application to three different test scenarios featuring irregular computational domains. The results are rigorously evaluated, and their precision and efficiency are effectively communicated through tables and figures.

The manuscript is well-written, and the results appear to be accurate and genuine. Prior to considering publication in this esteemed journal, the following comments should be addressed:

1. While the topic is intriguing and the paper holds the potential to contribute significantly to the literature, certain improvements are needed. It is imperative that the abstract clearly elucidates the problem studied, its significance, and the employed methodology. The paper contains several typos and errors, and foremost, a thorough revision of the English language is essential.
2. The Introduction should compellingly justify the study's utility and delineate its novelty or originality. This should involve discussing existing knowledge in the open literature and identifying gaps.
3. The rationale behind employing the presented technique for approximating solutions on both uniform and scattered nodes, leading to a sparse and well-conditioned coefficient matrix, should be clarified.
4. Further elaboration is required to explain why the technique approximates solutions on sets of uniform and scattered nodes, ultimately yielding a sparse and well-conditioned coefficient matrix.
5. The authors are encouraged to provide additional details regarding their original contributions throughout the manuscript.
6. It is recommended to ensure proper punctuation (comma or period) is consistently used at the end of equations, adhering to typing conventions. A comprehensive review of the entire paper for such errors is necessary.
7. Enhanced descriptions of the examples would enhance clarity.
8. Clear definition of symbols and proper equation numbering are essential.
9. The proposed methodology is promising in the other similar areas, the authors should mention some as future directions. (Doi: 10.1016/j.joes.2022.03.014; DOI: 10.1142/S0218348X22401429; DOI: 10.1016/j.jmrt.2021.07.029)
